# Genome sequences of two isolates of the yeast *Candida zeylanoides*: UCD849 from soil in Ireland, and AWD from an African wild dog

**DOI:** 10.1128/mra.01081-23

**Published:** 2024-02-09

**Authors:** Richard W. McLaughlin, Conor Hession, Sean Bergin, Aoife Cosgrove, Andrew Dowd, Niall Garvey, Gabrielle Litovskich, Evita Osaigbovo, Denis Popa, Cynthia Thuku, Geraldine Butler, Kenneth H. Wolfe, Kevin P. Byrne

**Affiliations:** 1 School of Liberal Arts and Sciences, Gateway Technical College, Kenosha, Wisconsin, USA; 2 School of Biomolecular and Biomedical Science, Conway Institute, University College Dublin, Dublin, Ireland; 3 School of Medicine, Conway Institute, University College Dublin, Dublin, Ireland; University of California Riverside, USA

**Keywords:** yeasts, genome analysis

## Abstract

We report genome sequences of two new isolates of the budding yeast *Candida zeylanoides*. Strain UCD849 from soil in Ireland was assembled into eight complete chromosomes. Strain AWD from an African Wild Dog in a US zoo was sequenced to draft level. The assemblies are 10.6 Mb and 99.57% identical.

## ANNOUNCEMENT

*Candida zeylanoides* is a species in the *Kurtzmaniella* clade of the budding yeast family Debaryomycetaceae (1). Strains have previously been isolated from sources including human (skin, throat, sputum, feces), animals (dog skin, dolphin skin), sea water, meats (salami, sausages, chilled beef), and plants (red clover) (1). It can be an opportunistic pathogen (2), and it has some biotechnological potential (3, 4).

*C. zeylanoides* UCD849 was isolated (Table 1) as part of an undergraduate research module (5). Soil material was passaged twice at room temperature in 9 mL liquid yeast extract-peptone-dextrose (YPD) containing chloramphenicol (30 µg/mL) and ampicillin (100 µg/mL) and cultured on YPD plates. The species was identified by PCR and Sanger sequencing of the ribosomal DNA internal transcribed spacer (ITS) and D1/D2 regions (accession numbers OR541107 and OR541115). Sequence identity was 100% (569/569 bp) in the ITS, and 99% (570/573 bp) in the D1/D2 region, to the type strain of *C. zeylanoides* (accession numbers KY102539 and NG_060834). DNA for genome sequencing was isolated from liquid YPD cultures grown at 30°C. For short-read sequencing, DNA was isolated by phenol/chloroform extraction. Illumina library construction (300-cycle v1.5 kit) and sequencing was done by Novogene (UK) Company Ltd. using a NovaSeq 6000 instrument with S4 flowcell and yielded 7.4 million read pairs (2 × 150 bp). Low-quality reads and adapter sequences were removed using Skewer (v0.2.2) (6). For long-read sequencing, DNA was extracted using a Biosearch Technology Masterpure yeast DNA purification kit (MPY80010). Oxford Nanopore (ONT) sequencing was done by combining two runs on a MinION MK1C instrument with flowcells FLO-MIN112 (R10.4) and FLO-MIN114 (R10.4.1) and barcoding kit SQK-NBD112-24. Raw data were basecalled (fast model) and demultiplexed using Guppy integrated in MinKNOW (v21.11) (ONT). NanoFilt (v2.3.0) (7) retained 63,110 reads with quality ≥7 and length ≥1,000 bp (reads N50 = 18,323 bp), which were then assembled using Canu (v2.0.0) (8), followed by five rounds of error correction using NextPolish (v1.4.1) with the Illumina reads (9). Default parameters were used for all software. Two gaps were closed manually: one in the rDNA array, and one that we closed using a 98 bp section of a contig from a *de novo* SPAdes (10) Illumina assembly. There is a single rDNA array located internally on chromosome 3 (accession number CP134677).

**TABLE 1:** Genome assembly statistics for *Candida zeylanoides* strains sequenced

Strain	UCD849	AWD
Isolated from	Soil collected in Kinnea, Co. Donegal, Ireland	Fecal material of an African Wild Dog (*Lycaon pictus*) living in the Great Plains Zoo & Delbridge Museum of Natural History, South Dakota, USA
GPS coordinates	55.271427, –7.474390	43.539202, –96.762399
Assembly size	10.68 Mb (nuclear) 27,483 bp (mitochondrial)	10.63 Mb
Assembly completeness	8 complete chromosomes + mitochondrial genome	92 contigs (>300 bp)
Telomere repeats	TGTATGGG (at all ends)	
N50	1.5 Mb	261 kb
Average nucleotide identity (ANI) (11) to type strain NRRL Y-1774 (12)	99.83%	99.59%
BUSCO (v5.1.2) genome completeness compared to Ascomycota lineage	92.9%	92.6%
G + C content	55.7%	55.7%

After isolation (Table 1) *C. zeylanoides* strain AWD was plated onto Sabouraud dextrose agar (Hardy Diagnostics) and incubated at 30°C for 48 h under aerobic conditions. Species identification was done using a MALDI Biotyper Sirius CA System (Bruker Daltonics) as outlined (13). For genomic DNA sequencing, *C. zeylanoides* AWD was grown aerobically at 30°C for 48 h on Trypticase soy agar (Hardy Diagnostics) and sent to SeqCenter (www.seqcenter.com). A ZymoBIOMICS DNA kit (Zymo Research) was used to isolate genomic DNA. Libraries were prepared using an Illumina DNA Prep kit (catalog 20060059) following the manufacturer’s recommendations, with custom IDT 10 bp unique dual indices. DNA was sequenced on an Illumina NextSeq 2000 instrument, which produced 17.9 million read pairs (2 × 151 bp). BCL Convert v4.0.3 (Illumina) was used for demultiplexing, adapter trimming, and quality control. The genome was assembled using Unicycler v0.5.0 (14).

For the genome assembly statistics of both strains see Table 1. Both isolates are available on request.

## Data Availability

These whole-genome shotgun sequences have been deposited at DDBJ/ENA/GenBank (BioProject accession number PRJNA1016273) with accession numbers CP134675-CP134683 (UCD849) (mitochondrial genome has accession number CP134683) and JAVRGF000000000 (AWD). The versions described in this paper are version 1. The raw reads have been deposited at SRA (accession numbers SRR26049581, SRR26049866, SRR26049867 and SRR26087802). The ITS and D1/D2 sequences of UCD849 are accession numbers OR541107 and OR541115
